# Modulation of mitochondrial activity in HaCaT keratinocytes by the cell penetrating peptide Z-Gly-RGD(DPhe)-mitoparan

**DOI:** 10.1186/s13104-018-3192-1

**Published:** 2018-01-30

**Authors:** Adam Richardson, Lewis Muir, Sasha Mousdell, Darren Sexton, Sarah Jones, John Howl, Kehinde Ross

**Affiliations:** 1 School of Pharmacy and Biomolecular Sciences, Liverpool John Moores University, Liverpool, L3 3AF UK; 2 School of Pharmacy, Faculty of Science & Engineering, University of Wolverhampton, Wolverhampton, WV1 1LY UK

**Keywords:** Cell penetrating peptides, Bioportides, Keratinocytes, Mitochondria

## Abstract

**Objective:**

Biologically active cell penetrating peptides (CPPs) are an emerging class of therapeutic agent. The wasp venom peptide mastoparan is an established CPP that modulates mitochondrial activity and triggers caspase-dependent apoptosis in cancer cells, as does the mastoparan analogue mitoparan (mitP). Mitochondrial depolarisation and activation of the caspase cascade also underpins the action of dithranol, a topical agent for treatment of psoriasis. The effects of a potent mitP analogue on mitochondrial activity were therefore examined to assess its potential as a novel approach for targeting mitochondria for the treatment of psoriasis.

**Results:**

In HaCaT keratinocytes treated with the mitP analogue Z-Gly-RGD(DPhe)-mitP for 24 h, a dose-dependent loss of mitochondrial activity was observed using the methyl-thiazolyl-tetrazolium (MTT) assay. At 10 μmol L^−1^, MTT activity was less than 30% that observed in untreated cells. Staining with the cationic dye JC-1 suggested that Z-Gly-RGD(DPhe)-mitP also dissipated the mitochondrial membrane potential, with a threefold increase in mitochondrial depolarisation levels. However, caspase activity appeared to be reduced by 24 h exposure to Z-Gly-RGD(DPhe)-mitP treatment. Furthermore, Z-Gly-RGD(DPhe)-mitP treatment had little effect on overall cell viability. Our findings suggest Z-Gly-RGD(DPhe)-mitP promotes the loss of mitochondrial activity but does not appear to evoke apoptosis in HaCaT keratinocytes.

## Introduction

Cell penetrating peptides (CPPs) have been studied extensively as vehicles that can traverse the plasma membrane to mediate intracellular delivery of bioactive cargoes including proteins, DNA and oligonucleotides [1, 2]. Most CPPs are inert molecules with cationic and hydrophobic residues that enable interactions with the cell surface and subsequent direct penetration into the cell or uptake via endocytosis [3]. Biologically active molecules associated with the CPP are taken up at the same time, enabling them to reach their targets.

Recently, exploitation of the intrinsic biological activity of selected CPPs has emerged as a promising strategy for targeting intracellular events [4]. Such bioactive CPPs, designated bioportides, modulate a range of processes, including angiogenesis, apoptosis and MAP kinase signalling [5]. Mastoparan, a bioportide from wasp venom, promotes cell death in cancer cell lines [6, 7] as does mitoparan (mitP), a mastoparan analogue with enhanced cellular penetration properties [8]. Furthermore, mitP specifically co-localises with mitochondria in astrocytoma and bladder cancer cells, leading to depolarisation, permeabilisation of the inner mitochondrial membrane and apoptosis [8, 9].

The use of CPPs for cutaneous delivery of macromolecules is emerging as a therapeutic approach for skin disorders [10]. However, the potential of bioportides themselves to treat cutaneous disorders has received limited attention. Nonetheless, the ability of mitP to promote apoptosis in a mitochondria-dependent manner parallels the mode of action of dithranol, an established topical therapy for the inflammatory skin disorder psoriasis [8, 11, 12]. Interestingly, modification of mitP with Z-glycine, the integrin-specific motif RGD and a D isomer of phenylalanine conferred enhanced potency on the peptide [8]. Therefore, in this study, we hypothesised that Z-Gly-RGD(DPhe)-mitP stimulates apoptosis in cultured keratinocytes. We found that Z-Gly-RGD(DPhe)-mitP treatment reduced mitochondrial activity and dissipated the mitochondrial membrane potential in HaCaT keratinocytes but did not appear to trigger apoptosis.

## Main text

### Methods

The reagents and procedures for preparation of CPP Z-Gly-RGD(DPhe)-mitP were as described [8] and 10 mmol L^−1^ stocks dissolved in water and stored − 20 °C until required. HaCaT keratinocytes were obtained and cultured as previously reported [13], seeded at 10,000/well of 96-well plates, and exposed to the indicated concentrations of Z-Gly-RGD(DPhe)-mitP for 24 h. The 3-(4,5-dimethylthiazol-2-yl)-2,5-diphenyltetrazolium bromide (MTT) reagent was purchased from Sigma-Aldrich (Gillingham, UK) and used in accordance with the manufacturer’s instructions, with absorbance measurements at 570 nm. The sulphorhodamine SRB assay was performed as described [14] and absorbance determined at 492 nm. For mitochondrial depolarisation studies, cells were stained with 1 μmol L^−1^ of the fluorescent dye JC-1 for 75 min at 37 °C, harvested by trypsinisation and analysed on an Accuri C6 flow cytometer. The Caspase-Glo 3/7 assay (Promega, Southampton, UK) was used according to the manufacturer’s instructions to monitor apoptosis and luminescence captured on a Clariostar plate reader (BMG Labtech, Aylesbury, UK).

*Statistical analyses* were performed in GraphPad Prism 5 (GraphPad Software, Inc., San Diego, CA) using unpaired t test with Welch’s correction which does not assume equal variances among the data.

### Results

#### Z-Gly-RGD(DPhe)-mitP alters mitochondrial activity

**Fig. 1:**
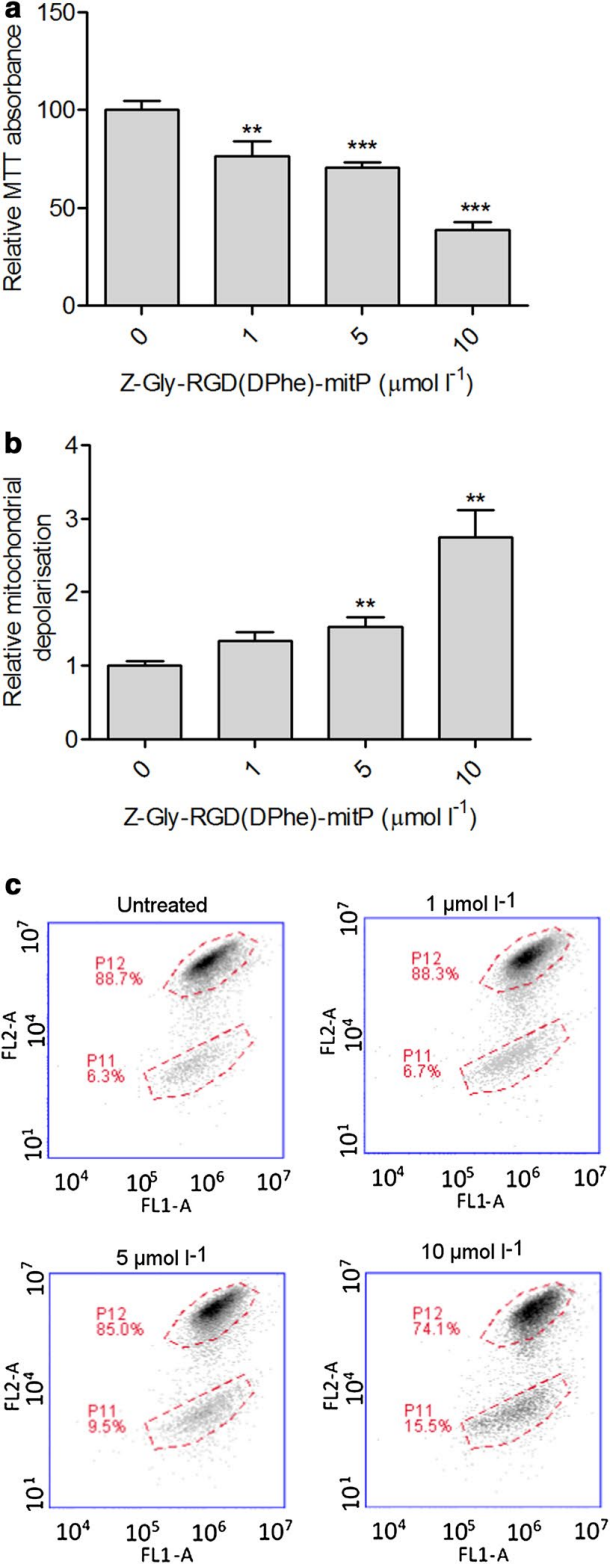
Effect of Z-Gly-RGD(DPhe)-mitP on HaCaT keratinocyte mitochondrial activity. **a** Cells were treated with Z-Gly-RGD(DPhe)-mitP for 24 h prior to evaluation of cell viability by MTT assay. **b** The proportions of cells depolarised after Z-Gly-RGD(DPhe)-mitP treatment, assessed by JC-1 staining and normalised to untreated cells. Data were pooled from three independent experiments performed in duplicate. **c** Representative data showing increase of cell population with mitochondrial depolarisation (P11) indicated by JC-1 staining after 24 h treatment with the indicated concentrations of Z-Gly-RGD(DPhe)-mitP. Data in shown in (**a**) and (**b**) are means + SEM; *p < 0.05, **p < 0.01, p < 0.0001; unpaired t test with Welch’s correction compared to the 0 μmol L^−1^ controls

For initial assessment of the impact of Z-Gly-RGD(DPhe)-mitP on HaCaT viability, mitochondrial activity was monitored using the MTT assay. Treatment with Z-Gly-RGD(DPhe)-mitP for 24 h led to a dose-dependent reduction in mitochondrial activity (Fig. 1a). At 10 μmol L^−1^, the CPP decreased MTT absorbance values to around 40% those of untreated cells. To determine whether loss of mitochondrial viability was associated with depolarisation mitochondria, JC-1 staining was performed. Flow cytometry analyses revealed a dose-dependent elevation of mitochondrial depolarisation (Fig. 1b, c) following a 24 h exposure to Z-Gly-RGD(DPhe)-mitP, with threefold higher levels of depolarisation in cells treated with 10 μmol L^−1^ of the CPP compared to untreated cells. Together, these results suggested that Z-Gly-RGD(DPhe)-mitP perturbed mitochondrial dynamics in HaCaT keratinocytes.

#### Effect of Z-Gly-RGD(DPhe)-mitP on caspase activity

**Fig. 2:**
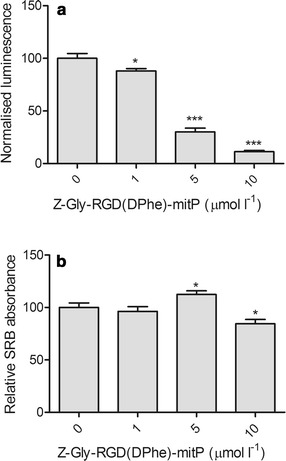
Alterations in caspase activity but not viability of HaCaT keratinocytes exposed to Z-Gly-RGD(DPhe)-mitP. Cells were treated with Z-Gly-RGD(DPhe)-mitP in serum-free medium for 24 h prior to (**a**) assessment of caspase 3/7 activity with a luminescence assay and (**b**) evaluation of cell density using the SRB assay. Data were pooled from three independent experiments performed in triplicate (**a**) or quadruplicate (**b**). Data shown are means + SEM; *p < 0.05; ***p < 0.0001 unpaired t test with Welch’s correction compared to the 0 μmol L^−1^ controls

To evaluate the ability of Z-Gly-RGD(DPhe)-mitP to promote keratinocyte apoptosis, caspase 3/7 activity was monitored using a luciferase-based assay. Contrary to expectations, basal caspase activity appeared to be reduced by Z-Gly-RGD(DPhe)-mitP (Fig. 2a). Cell viability was evaluated further using the SRB assay which monitors cell density based on the determination of cellular protein content. As shown in Fig. 2b, CPP treatment had little effect on absorbance readings, suggesting cell density was largely unaffected by Z-Gly-RGD(DPhe)-mitP. Thus the decline in mitochondrial activity (Fig. 1) or of caspase activity (Fig. 2a) does not appear to correlate with a general loss of cell viability as indicated by cellular protein content.

### Discussion

Despite recent progress in the provision of systemic therapy for severe psoriasis, topical anti-psoriasis regimens for mild-to-moderate disease remain largely unsatisfactory [15]. Resistance to apoptosis has long been identified as a feature of psoriatic keratinocytes, and the induction of apoptosis appears to underpin the mechanism of action of dithranol, an established topical for treatment for psoriasis [11, 16]. By dissipating the mitochondrial membrane potential and promoting cytochrome *c* release, the CPPs mastoparan, mitP and Z-Gly-RGD(DPhe)-mitP stimulate apoptosis in tumour cells [8]. These observations prompted us to considered the effects of Z-Gly-RGD(DPhe)-mitP on keratinocytes given that dithranol exerts its effects on these cells at least in part via similar mitochondrial mechanisms [11]. Our findings suggest that Z-Gly-RGD(DPhe)-mitP depolarised keratinocyte mitochondria as expected. However, the loss of mitochondrial bioactivity was uncoupled from the induction of caspase-mediated apoptosis in HaCaT keratinocytes. Indeed, Z-Gly-RGD(DPhe)-mitP appeared to decrease HaCaT basal caspase activity under the conditions used. Further studies are required in order to validate this putative anti-apoptotic effect of on Z-Gly-RGD(DPhe)-mitP on keratinocytes to determine relevance for skin disorders defined by extensive epidermal apoptosis, such as toxic epidermal necrolysis.

### Conclusions

The findings presented here indicate that Z-Gly-RGD(DPhe)-mitP dissipates the mitochondrial membrane potential of HaCaT keratinocytes without apparently inducing cell death. Further modification of Z-Gly-RGD(DPhe)-mitP or fusion to another peptide may thus be required for the development of a bioportide CPP as an alternative to dithranol.

## Limitations

One major limitation of the study is that absence of comparative caspase 3/7 data with known inducers of apoptosis. The Fas ligand (FasL) is known to induce HaCaT keratinocyte apoptosis as determined by nucleosomal DNA fragmentation [17]. However, we observed little effect on caspase 3/7 activity in HaCaT keratinocytes treated with FasL (data not shown). Therefore, additional measures of apoptosis, such as annexin V staining and DNA fragmentation are required. Furthermore, although the cell density SRB data do not suggest Z-Gly-RGD(DPhe)-mitP promotes cell death, it will be important to confirm that the loss of HaCaT keratinocyte caspase activity associated Z-Gly-RGD(DPhe)-mitP treatment is not due to necrosis. Finally, as the HaCaT keratinocyte cell line was used in this study, there remains a need to evaluate the impact of Z-Gly-RGD(DPhe)-mitP on mitochondria and apoptosis in primary keratinocytes and 3D epidermal models of psoriasis, and use transcriptome-wide approaches to fully define the biological processes associated with the effects of Z-Gly-RGD(DPhe)-mitP in human keratinocytes.

## Abbreviations

CPP: cell penetrating peptide
mitP: mitoparan
MTT: 3-(4,5-dimethylthiazol-2-yl)-2,5-diphenyltetrazolium bromide
SRB: sulforhodamine B
